# Snake Venom PLA_2_, a Promising Target for Broad-Spectrum Antivenom Drug Development

**DOI:** 10.1155/2017/6592820

**Published:** 2017-11-29

**Authors:** Huixiang Xiao, Hong Pan, Keren Liao, Mengxue Yang, Chunhong Huang

**Affiliations:** Department of Biochemistry, College of Basic Medical Sciences, Nanchang University, Nanchang, Jiangxi Province, China

## Abstract

Snakebite envenomation is a neglected global health problem, causing substantial mortality, disability, and psychological morbidity, especially in rural tropical and subtropical zones. Antivenin is currently the only specific medicine for envenomation. However, it is restricted by cold storage, snakebite diagnosis, and high price. Snake venom phospholipase A_2_s (svPLA_2_s) are found in all kinds of venomous snake families (e.g., Viperidae, Elapidae, and Colubridae). Along with their catalytic activity, svPLA_2_s elicit a wide variety of pharmacological effects that play a pivotal role in envenomation damage. Hence, neutralization of the svPLA_2_s could weaken or inhibit toxic damage. Here we overviewed the latest knowledge on the distribution, pathophysiological effects, and inhibitors of svPLA_2_s to elucidate the potential for a novel, wide spectrum antivenom drug targeting svPLA_2_s.

## 1. Introduction

Snakebite envenomation is a critical public health problem and fieldwork hazard, causing high mortality and morbidity, particularly in tropical and subtropical regions. As most ophidian incidents occur in rural areas of developing countries, accurate statistical data concerning the number of victims is difficult to obtain [1]. As extrapolated by Chippaux, worldwide 5,400,000 people are bitten by snakes, 2,500,000 are envenomed, 125,000 die, and more than 100,000 individuals suffer from severe sequelae each year [2]. Unfortunately, snakebite was neglected by governments and international health agencies for a long time, even though the snake bite mortality rate is equivalent to one-fifth of the deaths from malaria worldwide and half of the deaths from HIV/AIDS in India [3]. In 2009 the World Health Organization (WHO) recognized snake bite as a neglected tropical disease [1]. Currently, antivenin is the only specific treatment towards envenomation. Although the immunized animal sera (mainly horse or sheep) presently used are highly effective, they are limited by a few drawbacks [4]. First, local tissue damage resulting from snake venom exposure, often leading to amputation, cannot be reversed by antivenin [4]. Furthermore, early and late adverse reactions to antivenin (e.g., anaphylaxis, pyrogenic reactions, and serum sickness) occur in some cases [5]. Additionally, access to antivenins is often limited. Some remote, rural communities where antivenoms are most needed cannot get adequate supplies, due to the lack of cold chain storage and other complex political reasons. Finally, most antivenoms are too expensive for the patient's family in low-income countries [6].

Recently, the nonprofit French drug firm Sanofi Pasteur had ceased the production of Fav-Afrique, the most effective antivenin against Africa's vipers, mambas, and cobras. This has resulted in a large-scale snakebite crisis in rural Africa [7]. This alarming situation demonstrates the need for antivenin replacements and new antivenom drug candidates. This review article focuses on snake venom phospholipase A_2_s (svPLA_2_s), a chemical family that is widely distributed in venomous snake species. Here we describe svPLA_2_s, the antienvenomation effects of their inhibitors, and the potential of being a common target for broad-spectrum antivenom drugs.

## 2. Characteristics of svPLA_**2**_

Snake venoms are complicated mixtures, consisting of phospholipase A_2_s, metalloproteases, C-lectins, serine proteases, L-amino acid oxidases, disintegrins, and a few other compounds [1]. Most svPLA_2_s hydrolyze glycerophospholipids at the sn-2 position of the glycerol backbone, freeing lysophospholipids, and fatty acids. svPLA_2_s share 44–99% amino acid identity in their primarily structure, which results to high similarity in their tertiary structure [8]. Based on their size, location, function, substrate specificity, and calcium requirement, PLA_2_s are classified into six families. svPLA_2_ belongs to the secretory PLA_2_ (sPLA_2_) family (groups IA, IIA, and IIB) [9–11]. Cobras and kraits, rattlesnakes, and Gaboon vipers have svPLA_2_s in groups IA, IIA, and IIB, respectively [8]. There are also group IB enzymes which are mainly found in mammalian pancreas that have been reported in some snake venoms, such as* Oxyuranus scutellatus* [12],* Pseudonaja textilis* [13], and* Micrurus frontalis frontalis* [14]. These compounds are conserved in structure and have similar molecular masses (~10–20 kDa), 5–7 disulfide bonds, and analogous three-dimensional structures [15]. In Group I there are approximately 115–120 residues, 7 disulfide bonds (the unique disulfide linking residues 11 and 77), and G IA has a characteristic surface loop between residues 63 to 67 called elapidic loop [11]. While G IB has a five amino acids residues (residues 62–67) extension termed pancreatic loop, some G IB snake venom PLA_2_ even has an eight-residue propeptide segment in their mature state [13, 16]. In contrast, Group II has a C-terminal extension, the unique disulfide linking residues 50 and 137. GIIA have a 7-residue C-terminal extension and seven conserved disulfide bonds, while in Group IIB, the C-terminal extension is 6 residues, and only six disulfides remained in which a universally conserved 61–95 disulfide is lacking [11]. Furthermore, a new subgroup (Lys49 PLA_2_ homologues) can be created through mutation. Replacement of the 49th residue (asparagine) with lysine results in an inactive or weakly toxic PLA_2_. This lysine residue can also interact with other amino acids in the “calcium-binding loop” resulting in the loss of calcium-dependent catalytic activity [17, 18]. Most svPLA_2_s exist as monomers, but some exist in complexes, which mainly exhibit presynaptic neurotoxicity through combination of isoenzymes or other proteins [19].

## 3. PLA_**2**_s Are Extensively Distributed in Snake Venom

**Figure 1:**
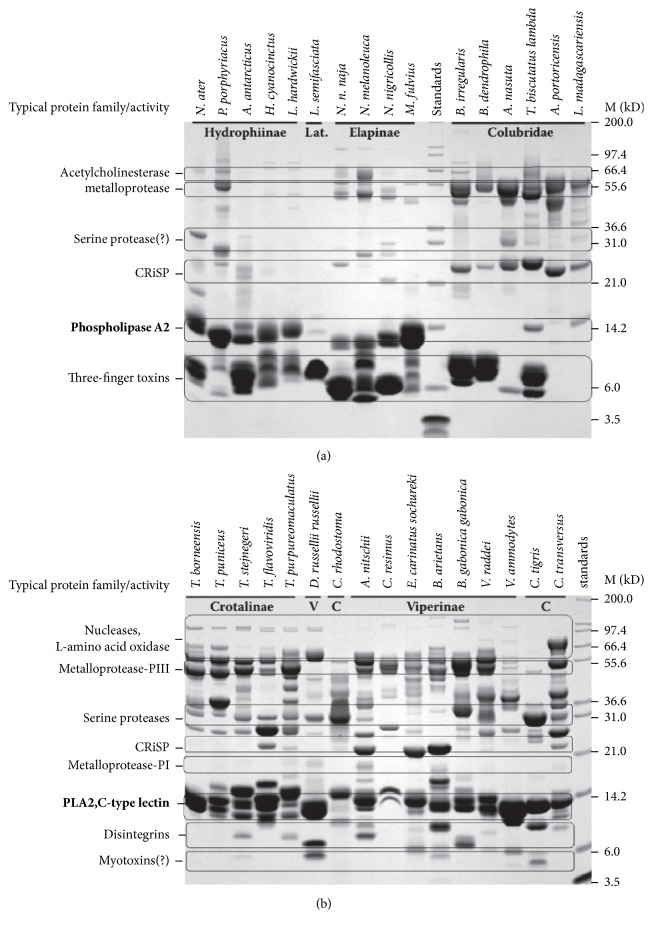
SDS-PAGE profile of major venom components in the main clades of venomous snakes (adapted from [20]). (a) Families: Elapidae, subfamilies Elapinae, Laticaudinae, Hydrophiinae, and Colubrinae. (b) Family: Viperidae, and subfamilies: Crotalinae (C) and Viperinae (V). Ovals enclose some bands that are typical of protein families, based on published mass. (?) indicates hypothetical protein family or activity.

Mackessy [20] analyzed crude venom from the main clades of venomous snakes via SDS-PAGE and found that svPLA_2_s existed in almost every family (Figure 1). The highest amounts were found in Elapidae, Viperidae, and Hydrophiidae. The lowest were found in Colubridae (which is usually nonvenomous). Through the application of transcriptomics and proteomics, we gained a better understanding of venom composition and the pharmacological properties of the venom components [21]. Betzel et al. found that PLA_2_s made up 32–59.8% in Viperidae snake venom [22]. However,* Bungarus fasciatus *venom was found to consist of up to 71% of PLA_2_s [23]. Moreover, Gutiérrez and Lomonte found that the most lethal fractions in* Micrurus fulvius *(family Elapidae) were two PLA_2_ molecules which represented 33.4% of the whole venom [24]. To date, more than 464 unique svPLA_2_s have been recorded in UniProtKB database. What has been presented above indicates that PLA_2_s are abundant and fatal toxins in most snake venoms.

## 4. svPLA_**2**_s Have a Wide Spectrum of Pharmacological Effects

Despite producing lysophospholipids and fatty acid proinflammatory mediators, svPLA_2_s also present a wide spectrum of pharmacological effects in victims, (i.e., neurotoxicity, myotoxicity, anticoagulant effects, cytotoxicity, cardiotoxicity, and edema, Table 1). The diverse toxic effects are tightly related to the multiple functional sites on the surface of svPLA_2_s and their different binding receptors [25].

### 4.1. svPLA_2_ Neurotoxicity

Neurotoxic svPLA_2_s can block neuromuscular transmission in vertebrate skeletal muscles causing acute neuromuscular weakness and paralysis resulting in respiratory depression and death [53]. Neurotoxic sPLA_2_s are mainly found in the Elapidae (kraits, elapids, and coral snakes) and Viperidae (vipers and rattlesnakes). Their toxicity varies greatly among species, ranging from 1 *μ*g/kg (Textilotoxin) to 380 *μ*g/kg (HDP-2 from* Vipera nikolskii*) [53]. Previous studies indicate that there is no correlation between toxicity and PLA_2_ hydrolysis activity. svPLA_2_ neurotoxicity affects presynaptic nerve terminals, so these compounds are commonly referred as presynaptic neurotoxins or *β*-neurotoxins (*β*-ntxs) [54]. *β*-ntxs are monomers or noncovalent complexes containing 2–5 subunits with at least one PLA_2_ subunit. To our knowledge, all *β*-ntxs hydrolyze phospholipids, especially anionic lipids (e.g., phosphatidylserine, phosphatidic acid, and phosphorylated phosphatidylinositols) which are abundant in the cytosolic leaflets of organelles and the plasma membrane of eukaryotic cells [55]. svPLA_2_s also bind to special tissue sites to achieve their neurotoxicity effects. The mechanism of svPLA_2_ neurotoxicity is still under investigation.

### 4.2. svPLA_2_ Myotoxicity

svPLA_2_s can induce acute necrosis of skeletal muscle (myonecrosis) [56]. In the envenomation, this myonecrosis can potentially lead to permanent tissue loss or amputation [57]. svPLA_2_ myotoxins are mainly found in venom from Elapidae, including sea snakes and Viperidae [58]. Depending on the venom, these svPLA_2_s can elicit local or systemic myotoxicity. Local myotoxicity is mainly elicited by viperid venom. This damage is limited to the region where the toxin is injected and is often coupled with hemorrhaging, blistering, and edema [57, 59]. Systemic myotoxicity is elicited by elapid venom (i.e., some sea snake, terrestrial elapids). This causes muscle damage and a distinct increase of creatine kinase (CK) activity in plasma and is associated with renal failure and myoglobinuria [58]. Along with sharing a highly conserved structure, svPLA_2_ myotoxins are tightly associated with neurotoxins. Both achieve a similar cellular lesion through membrane perturbation, cytosolic Ca^2+^ homoeostasis imbalance, and cell degeneration [60]. Furthermore, some neurotoxic svPLA_2_s (e.g., notexin and crotoxin) cause acute skeletal muscle necrosis, adding to systemic toxic effects (i.e., rhabdomyolysis) [60].

Residue 49 in myotoxic svPLA_2_s is usually associated with PLA_2_ enzymatic activity. Asp49-PLA_2_s are generally strongly catalytic whereas Lys49 homologues are either not catalytic or weakly catalytic. There are also other amino acid substitutions, such as Ser49, Arg49, Asn49, or Gln49 [56]. The lysophospholipids released from phospholipid that hydrolyzed by Asp49 PLA_2_ usually cause skeletal muscle necrosis via direct disruption of membrane stabilization and/or indirect biophysical alteration of membrane [61]. The Lys49 PLA_2_ myotoxins are devoid of catalytic activity, existing as homodimers in solution connected by noncovalent bonds [56]. Previous studies focused on the fact that amino acids composition of synthetic peptides has revealed that the C-terminal regions of 115–129 residues, which are positively charged and full of basic, aromatic, hydrophobic residues, are the key structure in eliciting myotoxic effects [62, 63]. Site-directed mutagenesis experiments proved that Tyr117, Arg118, Tyr119, Lys122, and Phe125 also have significant impacts on myotoxicity [64].

### 4.3. svPLA_2_ Anticoagulant Effect

The anticoagulant effect of svPLA_2_ usually causes bleeding in victim/prey by inhibiting one or two steps in the blood coagulation cascade. PLA_2_s can be classified as strong, weak, and nonanticoagulant based on the dose required to inhibit blood coagulation [65]. The hydrolysis of phospholipids by svPLA_2_ would be the primary mechanism to account for PLA_2_s' anticoagulation [66]. However, in the absence of phospholipids, some svPLA_2_s could also inhibit coagulation [67]. The correlation between svPLA_2_ enzymatic activity and anticoagulant effect is still unknown. Furthermore, there are other mechanisms that restrain coagulation, such as inhibition of the activation of the conversion of FX (blood coagulation factor X) to FXa (blood coagulation factor Xa) and/or prothrombin to thrombin [68].

svPLA_2_s can also induce other toxic effects such as myoglobinuria-inducing, hemolytic, and platelet aggregation initiating/inhibiting activities [49]. Their wide distribution, conserved structures, and various severe pharmacological effects suggest that svPLA_2_s represent a promising target for new antivenom medicine. Indeed, there is sufficient evidence that PLA_2_ inhibitors (PLIs) are effective in using snake venom envenomation therapy [69].

## 5. PLA_**2**_ Inhibitors Attenuate Morbidity and Mortality of Snakebite Envenomation

Due to the high cost, long production period, limited categories, short storage life, and common clinical side-effects of current antivenin, scientists have attempted to create antidotes from herbal extracts, marine compounds, mammalian and snake serum, and modified chemical molecules and peptides [70]. svPLA_2_s are the ideal target and widely used for antidote screening. Indeed, both natural and synthetic svPLA_2_ inhibitors are able to attenuate the morbidity and mortality of snakebite envenomation.

### 5.1. Natural svPLA_2_ Inhibitors from Plants, Marine Extracts, and Mammalian Serum

Medicinal plant extracts as traditional antidotes have long been used in countries where the urotherapy is unobtainable [71]. In addition, these traditional and herbal treatments are often used as adjuvant therapies along with the antivenin treatment. Most plant antitoxic agents function by neutralizing svPLA_2_'s toxicity. An active glycoprotein (WSG) from* Withania somnifera* completely inhibits the cytotoxicity, edema, and myotoxicity of NN-Xia-PLA_2_ isolated from* Naja atra* venom, but fails to neutralize the neurotoxicity [72–74]. WSG has a similar structure to the *α*-chain of the PLIs derived from Australian elapid serum and was found to interact with NN-XIa–PLA_2_, but the mechanism currently remains unknown [74].

The aqueous extract of* Casearia sylvestris* was found to be effective against two snake venom toxins (Asp49-PLA_2_ and Lys49-PLA_2_ isolated from venom of* B. moojeni*,* B. pirajai*,* B. neuwiedi*, and* B. jararacussu*). Indeed, this plant has been found to inhibit myotoxicity, hemorrhage, anticoagulation, and edema [75, 76]. It is also able to prevent myonecrosis initiated by two Lys49-PLA_2_ toxins (PrTX-I from* B. pirajai* and BthTX-I from* B. jararacussu* venom) and neuromuscular blockages [77]. Recently research has shown that human secretory PLA_2_ inhibitors (e.g., quercetin, biflavonoid morelloflavone [78, 79]) isolated from plant extracts can also inhibit svPLA_2_.

Marine organisms are also a reservoir for antivenoms. Manoalide (MLD), a natural product from sponge* Luffariella variabilis*, can irreversibly inhibit extracellular PLA_2_ activity of cobra and rattlesnake venom with an IC_50_ value of 1.9 and 0.7 *μ*M, respectively [80]. Its synthetic analogue, manoalogue (MLG), is also inhibitive to cobra PLA_2_ activity with an IC_50_ value of 7.5 *μ*M [81].

Natural svPLA_2_ inhibitors also exist in some mammalian serums. DM64 is an acidic glycoprotein isolated from serum of the opossum,* Didelphis marsupialis*. DM64 can completely prevent myofiber breakdown caused by myotoxins I (Asp49) and II (Lys49) of* B. asper* venom [82]. N-glycosylation sites (Asn46, Asn179, Asn183, and Asn379) in this antimyotoxic protein play important roles in this inhibitory action [83].

### 5.2. Snake Blood PLA_2_ Inhibitors

Many venomous and nonvenomous snake species are naturally resistant to the deleterious actions of snake venom components. In many cases, this is due to the presence of specific antitoxins circulating in their blood. These alexeteric factors are proteins generated in the snake's liver, with native molecular masses ranging from 75 to 180 kDa. These nonimmunoglobulin antitoxins are PLA_2_ inhibitors (i.e., snake blood phospholipase A2 inhibitors, sbPLIs) and are used to protect the snake from the internal or external envenomation.

sbPLIs can be produced by snakes of the Elapidae, Viperidae, Hydrophidae, Colubridae and Boidae families. These sbPLIs can be classified into three groups based on the homology of their amino acid sequence: *α*, *β* and *γ* [84]. Generally, the *α* and *γ* sbPLIs simultaneously occur in several snake species, while the *β*sbPLIs have only been reported in three snake species. When the target PLA_2_s are Lys49 homologues or Asp49 myotoxins, the sbPLIs are specifically called myotoxin inhibitor proteins (MIPs) [85, 86].

Since the first *α*PLI (BaMIP) was isolated from* B. asper* serum, 15 kinds of *α*sbPLIs have been discovered in the different venomous snake families. Previous studies have shown that BaMIP can block both myotoxins I and III (isolated from* B. asper* venom) [87]. The *α*PLIs, *α*TfPLI, and *α*AbsPLI also show good inhibition of the enzymatic activities of acid-PLA_2_ (isolated from Viperidae). CgMIP-II and AnMIP can inhibit the basic-PLA_2_ enzymatic activities of Viperidae venom. BaMIP, BmjMIP and BjussuMIP can inhibit the enzymatic activities and toxic effects (i.e., edema, myotoxicity, and cytotoxicity) of acid/basic-PLA_2_. Furthermore, Quirós et al. extracted a new myotoxin inhibitor *α*PLI from* A. nummifer* serum (AnMIP) and found that this protein, at a ratio of 1 : 1, could decrease 67% of the* A. nummifer* myotoxin II and 93% of the* B. asper *myotoxin I [85].

Currently four kinds of *β*sbPLIs have been found in three snake species. *β* PLI specifically inhibits the basic-PLA_2_ enzymatic activities of Viperidae. The first *β*sbPLI was purified from* G. brevicaudus* as a homotrimer and is specific for basic-PLA_2_s from homologous venoms and forms a stable PLA_2_-*β*sbPLI complex at a molar ratio of 1 : 1 [88].

Twenty-three types of *γ*sbPLIs have been found in venomous and nonvenomous species. *γ*PLI from Elapidae and other nonvenomous snakes can inhibit PLA_2_ activity in a range of different snake venoms. We recently reported a novel *γ*PLI isolated from the serum of* Sinonatrix annularis*, named *γ*saPLI, that showed a strong inhibition of lecithin degradation elicited by* D. acutus* venom PLA_2_s in an* in vitro* study [89]. The *γ*saPLI was also effective in the inhibition of hemorrhagic toxicities elicited by* D. acutus*,* N. atra, *and* A. halys* venom [90].

### 5.3. Poly or Monoclonal Antibodies of svPLA_2_ Are Effective in Neutralizing Snake Venom

Unlike the common antivenins of venom proteome, Garcia Denegri et al. developed a poly-antibody using a nontoxic PLA_2_ (BaSpII RP4) from* Bothrops alternatus *as antigen [91]. This antibody showed a specific and sensitive inhibition of the venom PLA_2_s' enzymatic activity. Furthermore, the myotoxicity and mortality of the crude venom were significantly reduced in the presence of anti-PLA_2_ IgG. When treated with a high dose of 2 × LD_50_, equivalent to 112 *μ*g of* B. alternatus* venom and 2.62 mg of IgG, all of the test animals survived after 48 h. In contrast, the control group (112 *μ*g venom preincubated with PBS) died within 4 hours. 5.25 mg of IgG treated animals could even endure as high as 4 times the LD_50_ dose of venom (224 *μ*g), with half of the treated group remaining alive at the end of 48 h. In contrast, the control group (224 *μ*g venom preincubated with PBS) died shortly within 90 mins.

Rodriguez et al. also produced a IgG against crotoxin (a basic PLA_2_), the principle toxin of* C. durissus terrificus *(C.d.t.) with high myotoxic and neurotoxic activities. Mice preincubated with the anticrotoxin IgG showed low mortality after 24 and 48 h of inoculation (at 4 *μ*g C.d.t. venom/test animal). The investigation showed that the IgGs of anti-PLA_2_ were more effective than anticrotalic serum at neutralizing lethal activity [92]. Additionally, the anti-PLA_2_ IgGs raised via immunization with P9a or P10a, two types of less toxic Cdt-PLA2s, cross-reacted with all the isoforms of PLA_2_s in the C.d.t. venom [93]. Although these antitoxic effects were only tested with their original venoms, the wide cross-reaction of these anti-PLA_2_ IgGs with other svPLA_2_s suggested that these compounds could likely also be used to neutralize other snake venoms. In other words, the improved neutralization activity of these anti-svPLA_2_ IgGs indicates svPLA_2_s are a promising target for broad-spectrum antivenom drug development.

### 5.4. Artificial Inhibitor of Mammal PLA_2_ Exhibits Effective Antivenom Activity

Varespladib (LY315920) was designed as an inhibitor of the IIa, V, and X isoforms of the mammalian secretory phospholipase A_2_ (sPLA_2_). This compound acts as an anti-inflammatory agent by disrupting the first step of the arachidonic acid pathway of inflammation. From 2006 to 2012, varespladib was under active investigation by Anthera Pharmaceuticals for using as a potential therapy for several inflammatory diseases, including acute coronary syndrome and acute chest syndrome [94, 95]. Thought to be an effective antiatherosclerotic agent, varespladib showed promising therapeutic effects in reducing plasma sPLA_2_ and low-density lipoprotein (LDL) [96].

Varespladib has recently been repurposed as an effective broad-spectrum svPLA_2_ inhibitor and used for treatment of snakebite envenomation. Varespladib and its orally bioavailable prodrug methyl-varespladib (LY333013) showed strong inhibitory ability of 28 kinds of svPLA_2_s from six continents. Indeed, the IC_50_ values ranged from nano- to picomolars in an* in vitro* experiment [97]. Additionally, the compound elicited surprising effects with eastern coral snake* (Micrurus fulvius)* venom, which was considered to have the highest sPLA_2_ activity and most intense hemo- and neurotoxic effects. Pretreatment with 0.1 mg of varespladib prolonged survival in mice at 4 times the LD_50_ dose of eastern coral snake venom over the course of 8 h. All the negative control mice died at an average of 63 min, whereas the varespladib treatment group survived for an average of 1140 min. Varespladib also showed promising* in vivo* protection in* Vipera berus *envenomed mice. Mice treated with a subcutaneous injection of a 100% lethal dose of venom and varespladib survived for more than 24 h [97]. These findings are solid evidence of svPLA_2_ being the target for a broad-spectrum antivenom.

## 6. Conclusions

svPLA_2_s are widely distributed in snake venoms. A svPLA_2_ could elicit one or more pharmacological effects (e.g., neurotoxicity, myotoxicity, anticoagulant, and edema). Furthermore, svPLA_2_s can interact with other svPLA_2_s (e.g., two different svPLA_2_s, the “Asp” and “Lys” myotoxins from* Bothrops asper*, have been shown to synergistically enhance myonecrosis in* in vitro* and* in vivo* studies [98]) or other venom components (e.g., taicatoxin, a Ca^2+^ channel inhibitor composed of an *α*-neurotoxin-like peptide, a neurotoxic phospholipase A_2_, and a serine protease inhibitor, connected by noncovalent bonds [99]).

A variety of PLA_2_ inhibitors were discovered or synthesized in the past few decades. Most inhibitors extracted from medical plants, marine animals, and mammalian serum specially inhibit svPLA_2_ toxicity. sbPLIs are natural, endogenous protective components against snake venom, among which the *γ*PLI were commonly inhibitive to different category of venoms [100]. Anti-PLA_2_ antibodies could specifically inactivate enzymatic activity and toxicity, both with the original venom and other svPLA_2_s [93]. Indeed, some of these compounds could function even better than the antivenin that is currently clinically applied [92]. A synthetic human sPLA_2_ inhibitor varespladib was found to possess the ability to neutralize a variety of snake venoms worldwide, with significant prolongation of survival time on rats that were inoculated with varespladib simultaneously or following exposure [97]. In conclusion, the anti-PLA_2_ drugs are promising antidotes for a broad-spectrum of snake venoms and other animal toxins and could also be effective in prevention of inflammatory reactions (i.e., systemic toxicological syndromes).

## Figures and Tables

**Table 1:** Features, toxicities, binding receptors, and enzymatic activity of snake venom PLA_2_s.

Name	Snake species	Structural features subtype^a^	Toxicities	Lethality in mouse (*μ*g/kg)^b^	Binding proteins in tissue^c^	PLA_2_ activity (*μ*mol/min/mg toxin)^d^	Reference
*Neurotoxin*							
Crotoxin	*Crotalus durissus terrificus*	Heterodimeric; A: IIA-sPLA_2_-like	Neurotoxicity; myotoxicity; cardiotoxicity	60–240 (i.v.)	Crocalbin; CaM	85	[26]
		B: IIA-sPLA_2_					
MsPLA_2_-I	*Micrurus spixii*	Monomeric; IA-PLA_2_	Neurotoxicity; myotoxicity; antiplasmodial activity; edema	n.d.	nAchR	Yes	[27]
Taipoxin	*Oxyuranus scutellatus*	Trimeric; *α*: IA, toxic; *β*: IA- sPLA_2_like; *γ*: IB-sPLA_2_; glycosylated	Presynaptic neurotoxicity;	2 (i.v.)	M-sPLA_2_R; NP; TCBP-49	0.4	[28–30]
			cytotoxicity				
Textilotoxin	*Pseudonaja textilis*	Pentameric; A, B and C are IA- sPLA_2_; D_2_, identicalS-SlinkedIB-sPLA_2_s, glycosylated	Presynaptic neurotoxicity	1 (i.v.)	M-sPLA_2_R	3.2	[13, 28, 31]
Ammodytoxin	*Vipera ammodytes*	Monomeric; IIA-sPLA_2_	Presynaptic neurotoxicity;	21 (i.v.)	M-sPLA_2_R; CaM; PDI; FXa; 14-3-3 proteins	280	[32–35]
			anticoagulant				
*β*-Bungarotoxin	*Bungarus multicinctus*	Dimeric; A: IA-sPLA_2_	Presynaptic neurotoxicity	19–130 (i.p.)	v.-d. K^+^ channel	61	[36, 37]
		S-S linked to subunit B: BPTI-like					
Notexin	*Notechis scutatus*	Monomeric; IA -sPLA_2_ (Asp49)	Myotoxicity; presynaptic neurotoxicity; nephrotoxicity	17 (i.v.)	n.d.	1390	[38, 39]
*Myotoxin*							
Myotoxin III	*Bothrops asper*	Dimeric; IIA -sPLA_2_ (Asp49)	Myotoxicity;	470 (i.v.)	n.d.	750	[40]
			anticoagulant; edema				
Myotoxin II	*B. moojeni*	Monomeric; IIA-sPLA_2_ (Lys49)	Myotoxicity; edema	7600 (i.p.)	n.d.	None	[41]
CoaTx-II	*Crotalus oreganus abyssus*	Dimeric; IIA-sPLA_2_ (Lys49)	Myotoxicity; edema; antibacterial activity	n.d.	n.d.	None	[42]
Cr,5	*Calloselasma rhodostoma*	Monomeric; IIA-sPLA_2_ (Lys49)	Cytotoxicity; myotoxicity; edema	70 (i.c.v.)	n.d.	None	[43]
BaTX	*Bothrops alternatus*	Monomeric IIA-sPLA_2_ (Lys49)	Cytotoxicity; myotoxicity; edema; neurotoxicity	7000 (i.v.)	n.d.	None	[44]
Cr-IV 1	*Calloselasma rhodostoma*	Monomeric; IIA-sPLA_2_ (Asp49)	Myotoxicity; cytotoxicity; edema	70 (i.c.v.)	n.d.	0.014	[45]
Ammodytin L	*Vipera ammodytes*	Monomeric; IIA-sPLA_2_ (Ser49)	Myotoxicity	3600 (i.p.)	n.d.	None	[46]
*Anticoagulant*							
Daboxin P	*Daboia russelii*	Monomeric; IA-sPLA_2_	Strong anticoagulant	n.d.	FX; FXa	1140	[47]
RVV*-*PFIIc′	*D. russelii*	Monomeric; IIA-sPLA_2_ (Asp49)	Anticoagulant	100 (i.p.)	n.d.	Yes	[48]
CM-IV	*Naja nigricollis*	Monomeric; IIA-sPLA_2_ (Asp49)	Strongly anticoagulant; presynaptic neurotoxicity	180 (i.p.)	FXa; FVIIa	Yes	[49, 50]
CM-II	*Naja mossambica*	Monomeric; IA-sPLA_2_	Weak anticoagulant; myotoxicity; neurotoxicity	n.d.	TF; FVII	Yes	[51, 52]

^a^BPTI, bovine pancreatic trypsin inhibitor; ^b^i.c.v., intracerebroventricular; i.v., intravenous; i.c., intracisternal; i.p., intraperitoneal. n.d.: not determined; ^c^CaM, calmodulin; NP, neuronal pentraxin; PDI, protein disulfide isomerase; TCBP-49, taipoxin-associated calcium-binding protein 49; M-sPLA_2_R, M-type sPLA_2_ receptor. Fxa, blood coagulation factor Xa; FX, blood coagulation factor X; TF, tissue factor; FVII, blood coagulation factor VII; FVIIa, blood coagulation factor VIIa; v.-d. K^+^ channel, voltage-dependent K+ channels; ^d^phospholipase A_2_ activity is in *μ*mol/min/mg of toxin; Yes, original research paper does not show phospholipase A_2_ activity in concrete number or not in *μ*mol/min/mg of toxin; None, all PLA_2_ homologues are here considered to be enzymatically inactive. Adapted from [50, 51].
